# Characterization of the "*deqi*" response in acupuncture

**DOI:** 10.1186/1472-6882-7-33

**Published:** 2007-10-31

**Authors:** Kathleen KS Hui, Erika E Nixon, Mark G Vangel, Jing Liu, Ovidiu Marina, Vitaly Napadow, Steven M Hodge, Bruce R Rosen, Nikos Makris, David N Kennedy

**Affiliations:** 1 Athinoula A. Martinos Center for Biomedical Imaging, Department of Radiology, Massachusetts General Hospital and Harvard Medical School, Boston, MA, USA; 2 School of Medicine, Case Western Reserve University, Cleveland, OH, USA; 3 Center for Morphometric Analysis, Department of Neurology, Massachusetts General Hospital and Harvard Medical School, Boston, MA, USA

## Abstract

**Background:**

Acupuncture stimulation elicits *deqi*, a composite of unique sensations that is essential for clinical efficacy according to traditional Chinese medicine (TCM). There is lack of adequate experimental data to indicate what sensations comprise *deqi*, their prevalence and intensity, their relationship to acupoints, how they compare with conventional somatosensory or noxious response. The objective of this study is to provide scientific evidence on these issues and to characterize the nature of the *deqi phenomenon *in terms of the prevalence of sensations as well as the uniqueness of the sensations underlying the *deqi *experience.

**Methods:**

Manual acupuncture was performed at LI4, ST36 and LV3 on the extremities in randomized order during fMRI in 42 acupuncture naïve healthy adult volunteers. Non-invasive tactile stimulation was delivered to the acupoints by gentle tapping with a von Frey monofilament prior to acupuncture to serve as a sensory control. At the end of each procedure, the subject was asked if each of the sensations listed in a questionnaire or any other sensations occurred during stimulation, and if present to rate its intensity on a numerical scale of 1–10. Statistical analysis including paired t-test, analysis of variance, Spearman's correlation and Fisher's exact test were performed to compare responses between acupuncture and sensory stimulation.

**Results:**

The *deqi *response was elicited in 71% of the acupuncture procedures compared with 24% for tactile stimulation when thresholded at a minimum total score of 3 for all the sensations. The frequency and intensity of individual sensations were significantly higher in acupuncture. Among the sensations typically associated with *deqi*, aching, soreness and pressure were most common, followed by tingling, numbness, dull pain, heaviness, warmth, fullness and coolness. Sharp pain of brief duration that occurred in occasional subjects was regarded as inadvertent noxious stimulation. The most significant differences in the *deqi *sensations between acupuncture and tactile stimulation control were observed with aching, soreness, pressure and dull pain. Consistent with its prominent role in TCM, LI4 showed the most prominent response, the largest number of sensations as well as the most marked difference in the frequency and intensity of aching, soreness and dull pain between acupuncture and tactile stimulation control. Interestingly, the dull pain generally preceded or occurred in the absence of sharp pain in contrast to reports in the pain literature. An approach to summarize a sensation profile, called the *deqi composite*, is proposed and applied to explain differences in *deqi *among acupoints.

**Conclusion:**

The complex pattern of sensations in the *deqi *response suggests involvement of a wide spectrum of myelinated and unmyelinated nerve fibers, particularly the slower conducting fibers in the tendinomuscular layers. The study provides scientific data on the characteristics of the *'deqi' *response in acupuncture and its association with distinct nerve fibers. The findings are clinically relevant and consistent with modern concepts in neurophysiology. They can provide a foundation for future studies on the *deqi *phenomenon.

## Background

Acupuncture stimulation elicits *deqi*, a composite of unique sensations interpreted as the flow of *qi *or 'vital energy'. This state is essential for clinical efficacy according to traditional Chinese medicine (TCM) [1]. Understanding this phenomenon in modern biomedical terms is therefore important for elucidating the mechanisms of acupuncture action. However, among the constellation of sensations that can be elicited by different acupuncture techniques, there is lack of adequate experimental data to indicate what sensations comprise *deqi*, their prevalence and intensity, their relationship to acupoints, needling techniques and nerve fiber systems, and how they compare with conventional somatosensory or noxious stimuli. This study aims to explore some of these fundamental issues and define the characteristics of *deqi *response in acupuncture. It has been demonstrated that many of the *deqi *sensations are conveyed by different nerve fiber systems. Aching, soreness, distension, heaviness, warmth and dull pain are conveyed by the slower conducting Aδ and C fibers, whereas numbness is conveyed by the faster conducting Aβ fibers [2-4]. Since the Aδ and C fibers are more densely distributed in the tendinomuscular layers [5,6], one may predict that acupuncture needle manipulation at this depth will elicit more aching, soreness, fullness and dull pain than superficial tactile stimulation. These differences in the pattern of psychophysical response can be used to discriminate between acupuncture and conventional touch stimulus. This postulate was put to test in the present study.

The data were collected as part of a larger project using fMRI imaging to study the effects of acupuncture on the human brain. Manual acupuncture was performed on 42 healthy adult volunteers at LI4 (*Hegu*), ST36 (*Zusanli*) and LV3 (*Taichong*), three commonly used acupoints in traditional Chinese acupuncture. The sensations reported by the subjects provide the data used in this report. The list of sensations categorized as *deqi *was based on the descriptors provided in TCM literature and reports by patients in clinical practice [1]. Sharp pain was regarded to result from inadvertent noxious stimulation rather than acupuncture *deqi*, as evidenced by their distinct differences in hemodynamic response by fMRI [7,8]. As the objective of the study was to characterize the nature of the *deqi phenomenon*, detailed statistical analysis was performed to characterize the prevalence of sensations as well as the uniqueness of the *deqi *experience. Furthermore, we explored the characteristics of sensations as a '*deqi composite*', a single-valued summary of the reported simultaneous sensations. This index can be used as a covariate in the future exploration of the hemodynamic response of the brain to acupuncture demonstrated by fMRI and its correlation with the efficacy of acupuncture in clinical practice. It should be noted that this report is devoted to the psychophysical response in the study; its relationship to the hemodynamic response in the brain will follow in a separate communication.

## Methods

The study was conducted at the Athinoula Martinos Center for Biomedical Imaging at Massachusetts General Hospital and Medical School. The list of sensations categorized as *deqi *was based on the descriptors provided in TCM literature and reports by patients in clinical practice [1] rather than descriptors based on questionnaires for pain studies. Sharp pain was regarded to result from inadvertent noxious stimulation rather than acupuncture *deqi*, as evidenced by their distinct differences in hemodynamic response by fMRI [7,8]. Statistical analysis including paired t tests, ANOVA, Spearman's correlation and Fisher' exact test was performed to demonstrate the prevalence of the sensations as well as the uniqueness of the *deqi *experience.

### Subjects

The study was performed on 20–47 years old (29.0 +/- S.D 7.8), right-handed, acupuncture naïve healthy adult volunteers, 15 male and 27 female, 32 Caucasians, 6 Asians, 2 Hispanics and 2 Africans with informed consent, as approved by the Massachusetts General Hospital Subcommittee on Human Studies. The subjects were screened and excluded for major medical illnesses, history of head trauma, neuropsychiatric disorders, use of medications within one week, and contraindications for exposure to a high magnetic field. The sample size was determined by the minimum number of subjects necessary to detect activation/deactivation differences comparable to what had been observed in our previous fMRI studies, with 80% power.

### Procedures

The subjects were blinded to the procedures and could not see the sites undergoing stimulation from their supine position in the scanner. They were told that the acupuncture performed at different acupoints with different techniques would generate different needling sensations. Tactile (touch) stimulation was performed prior to acupuncture when the subjects were still naïve to acupuncture as a sensory comparison for the acupuncture stimulation. Thus, the comparison stimulation also took into account expectation and its placebo effects. Tactile stimulation and acupuncture were both performed at the same acupoint in 16 subjects each for LI4 and ST36, and 13 subjects for LV3. Three of the 42 subjects received tactile stimulation and acupuncture at all three acupoints; the remaining 39 subjects received acupuncture to all three acupoints, but only the paired tactile stimulation to the first of their acupoints. Analyses comparing tactile stimulation to acupuncture stimulation were performed on the paired sensory – acupuncture datasets. Data from all 3 acupoints for each subject was used in the Spearman's correlation of intensities of sensations in acupuncture.

**Figure 1:**
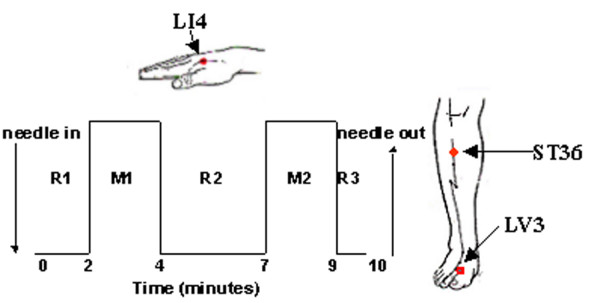
**Experimental paradigm. Manual acupuncture was administered to LI4, LV3 and ST36 on the right**. The subject's sensitivity to needling was pre-tested and adjusted to tolerance prior to scanning. After remaining in place for 2 min (R1), the needle was rotated forward and backward with even motion for 2 min at the rate of 60 times per minute with a amplitude of approximately 180° in each direction (M1). After a rest period of 3 min (R2), needle manipulation was repeated in like manner (M2). The needle was withdrawn 1 minute after completion of acupuncture. For tactile stimulation control, the acupoint was tapped with a size 5.88 von Frey monofilament using a matched paradigm.

Acupuncture and tactile stimulation control was delivered to LI4 on the hand, LV3 on the foot and ST36 on the lower leg on the right in randomized order by an acupuncturist with over 25 years of clinical experience (JL). The individual's sensitivity to needle manipulation was pretested, aiming to elicit *deqi *sensations without noxious pain. The stimulation paradigm is depicted in Figure 1. The needle was rotated approximately 180° in each direction with even motion at the rate of 60 times/min for 2 min during M1 and M2. The needle remained in place during the rest periods R1, R2 and R3. Each procedure lasted a total of ten minutes. In order to avoid excess discomfort, the subject was instructed to raise one finger if any sensation reached the intensity of 7–8 on a scale of 1–10 and 2 fingers in case of any sharp pain. When so signalled, the acupuncturist would adjust the force of stimulation so that the sharp pain would disappear within a few seconds. The acupuncture stimulation procedure was performed twice for each acupoint. Sterile, one-time use only stainless steel needles were used for LV3 (0.20 mm diameter) and ST36 (0.22 mm diameter) (KINGLI Medical Appliance Co. Wuxi, China). Silver needles (0.23 mm diameter) were used for LI4 (Matsuka, Tokyo, Japan). Superficial tactile stimulation was performed by gentle tapping with a size 5.88 von Frey monofilament, a standard method of sensory stimulation, prior to acupuncture with needling. The purpose of this design was to explore how acupuncture sensations might differ from the sensations elicited by the conventional sensory stimulus of touch. At the end of each tactile stimulation or acupuncture procedure, the subject was questioned by another researcher in the team if each of the *deqi *sensations (aching, pressure, soreness, heaviness, fullness, warmth, cooling, numbness, tingling, dull pain), sharp pain or any other sensations occurred during the stimulation, and to rate its intensity on the scale of 1–10 (1–3 mild, 4–6 moderate, 7–9 strong, 10 unbearable).

### Data analysis

The analyses were performed on each dataset from an acupuncture or tactile stimulation procedure. The paired t tests, ANOVA and Fisher's exact tests were performed on the average of the duplicate datasets for the acupuncture and tactile stimulation. Spearman's correlation for intensities of different sensations was performed on the individual datasets with acupuncture stimulation (41, 40, 41 datasets for LI4, ST36 LV3 respectively). The data were analysed both as continuous measures of a sensation, and also as a binary indication of its presence or absence. A sensation was determined to be present if the reported level, averaged over replicates, reached a minimal score of 1.

The datasets were divided into 3 groups 1) *deqi*, 2) mixed (*deqi *+ sharp pain), and 3) no sensations (neither *deqi *nor sharp pain) according to the sensations recorded at the end of each experimental procedure. None of the participants experienced acute pain without *deqi*. While dull pain was included as an important component of *deqi*, sharp pain of different forms such as stabbing, burning or pricking, was regarded as inadvertent noxious stimulation, and the co-occurrence of sharp pain with *deqi *was classified as a 'mixed response'. This scheme of categorization was based on the distinct differences in the hemodynamic response of the brain to these two categories of psychophysical response as evidenced by neuroimaging in prior studies. The pain neuromatrix was inhibited in *deqi *but activated in the presence of sharp pain [7,8].

### Characterization of the *deqi *response

We characterized the sensory responses elicited by the stimulation paradigm through a number of statistical approaches as described below. The set of observations and analyses we present are organized in support of three objectives: a) characterization of the prevalence of sensations elicited by the acupuncture stimulation; b) characterization of the uniqueness of the sensations associated with the *deqi *experience; and c) exploration of characteristic of sensations that could be used as a '*deqi composite*', a single-valued summary of the reported simultaneous sensations. Each of these objectives was explored using techniques as described below.

### Prevalence

#### Frequency of overall response groups

Fisher's exact test was conducted on the data from individual acupoints and on the data pooled from all points. We first tested for differences in the overall response (i.e. *deqi*, mixed, or no sensations) in acupuncture *versus *tactile stimulation control. There is as yet no consensus in regard to the number of sensations or magnitude of response to define the overall sensory experience as a *deqi *response. We have set two thresholds based on the sum of the scores for all sensations: T = 1, a minimal experience of sensations; and T = 3, a more stringent requirement. The threshold selected for data analysis will depend on the purpose of the analysis.

#### Frequency of sensations

We compared the frequency of sensations that reached the thresholds within the same subjects, between an acupoint and the corresponding tactile stimulation control. In order to do this, we tabulated the presence or absence of a sensation for each of the 10 sensations separately, with acupuncture *versus *the corresponding tactile stimulation control for each acupoint.

#### Intensity of sensations

We compared the mean sensation intensity between acupuncture and tactile stimulation control separately for each acupoint using paired t-tests. Simple paired t-tests using all of the data would be unreliable, due to the many zero scores reported for a number of the sensations. We therefore performed the t-tests on mean sensations only for subjects who reported a sensation above the threshold of 1 in acupuncture regardless of its presence or absence in tactile stimulation control. Thus, the p-values are for comparison of the intensity for acupuncture *vs*. tactile stimulation, conditional on the subject experiencing measurable sensation during acupuncture.

#### Correlation between the intensities of sensations

Spearman's correlation was performed on normalized data of all acupuncture procedures to determine if a correlation existed between the intensities of the different sensations that occurred during acupuncture for each acupoint, conditioned that the sensation was experienced at the minimal total score of 1.

### Uniqueness

#### Specificity of individual sensations for acupuncture *vs*. tactile stimulation

This analysis tests the null hypothesis in statistics that specific individual sensations co-occur in acupuncture and tactile stimulation. Rejecting this hypothesis informs us that if a subject felt a sensation in acupuncture then they did not feel it in tactile stimulation or *vice versa*. This comparison was performed by Fisher's exact test, pooled and for each acupoint. The reason for doing the test both ways is that the use of data pooled from all acupoints assumes that any association is independent of acupoint, but we will present evidence below to suggest that the nature of *deqi *is probably different for different acupoints. Doing Fisher's exact test separately for each acupoint avoids the independence assumption, but this approach might suffer from lack of power due to the small counts for certain sensations.

#### Comparison of the frequency and the intensity by which acupuncture exceeds tactile stimulation control for each sensation between acupoints

We applied Fisher's exact test to all datasets to determine the differences in frequency between acupuncture and tactile stimulation control, for each sensation, between the acupoints. We applied ANOVA to test for the differences in intensity between acupuncture and tactile stimulation control, for each sensation, between the acupoints. The ANOVA analysis was applied to the thresholded data that only included subjects who experienced that sensation during acupuncture as well as to all datasets.

### *Deqi *composite metric

#### Rank ordering of individual sensations

One difficulty in comparing combinations of sensations between groups and between experimental conditions is that a sensation profile is 10-dimensional. Hence a very large number of subjects would be required to adequately and reliably explore this high-dimensional space of sensation combinations. It is very desirable to reduce a sensation profile to a single number, a '*deqi composite*', which can then be used to characterize and compare *deqi *using standard univariate statistical procedures. We explored reduction of the set of sensations to a single value as follows: (1) for each subject determine the mean difference in intensity between acupuncture and tactile stimulation control for each sensation/acupoint combination; (2) average these differences over subjects; and (3) normalize the averaged differences so that the sum over sensations equals 1. The sets of values thus defined will be referred to as '*deqi weights*'.

#### '*Deqi *composite': differentiation of sensation and acupuncture stimulation

Given the *deqi *weights defined above, we can ask if using the observed patterns of sensations, the weighted index, '*deqi composite*' calculated by a summation over all sensations of each sensation score multiplied by its weight provides additional evidence for differentiation between the tactile and acupuncture stimulation conditions. ANOVA was used to discriminate acupuncture from tactile stimulation and to test differences between the acupoints, using both the 'simple' average and the 'weighted' average of the *deqi *sensations.

## Results

Due to the complexities of the analyses and results, we provide a summary of the major findings below. The findings are organized in the same order as their counterparts in the *Data Analysis *subsection of *Methods *that describes the statistical analyses used to obtain these results.

### Summary of results

Overall *deqi *response was significantly more frequent in acupuncture than in tactile stimulation control. In terms of sensation prevalence, the frequency of individual sensations was significantly higher in acupuncture than in tactile stimulation control. In addition, acupuncture elicits a unique set of sensations, as the occurrence of each sensation (with the exception of tingling) was distinct between acupuncture and tactile stimulation for each subject. Significant differences were found in the intensity of sensations between acupuncture and tactile stimulation control. Dull pain was significantly more intense in LI4 than in ST36. Aching, soreness and pressure appeared to be most important for the characterization of *deqi*. Use of the weighted average appeared to provide greater power in detection of the sensations specific to the *deqi *experience. All three points demonstrated correlation between aching and soreness, between heaviness and pressure, between dull pain and aching or soreness, and between tingling and numbness.

### Detailed Results

#### Frequency of the overall *deqi *response

**Figure 2:**
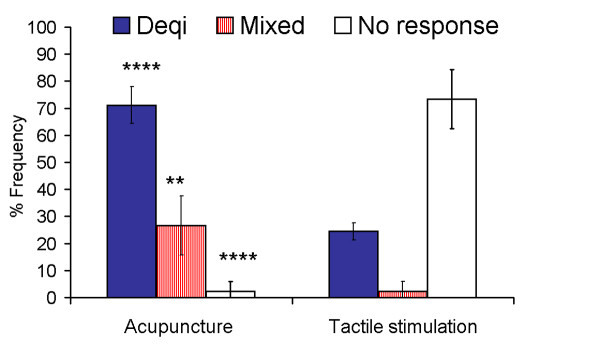
**Comparison of the frequency of different categories of psychophysical responses between acupuncture and tactile stimulation control**. When thresholded at a score of 3, the pooled data (N = 45) from the three acupoints showed a *deqi *frequency of 71% for acupuncture compared with 24% for tactile stimulation control (p < 0.0001). Standard error bars are based on a 95% Confidence Interval. Fisher's exact test. **p < 0.01, ****p < 0.0001. For more details on data refer to Table 1.

For the pooled data from all acupoints, the three categories of overall response demonstrated significant differences between acupuncture and tactile stimulation at both thresholds used for the definition of *deqi *(Figure 2, Table 1). Of the two thresholds, the minimal score of 1 may be too lenient a criterion for designating a response as *deqi*. When thresholded at T = 3, the pooled data showed a *deqi *frequency of 71% for acupuncture compared with 24% for tactile stimulation control (p < 0.0001). The frequency was slightly higher at LI4 and ST36 (76%) than at LV3 (61%). The *deqi *sensations were accompanied by brief occurrences of mild to moderate sharp pain in 27% of all acupuncture procedures. In tactile stimulation control, gentle tapping with a nylon filament caused pain in only 2% of subjects. The most striking contrast between tactile stimulation and acupuncture was the frequent failure to elicit any *deqi *sensations in tactile stimulation (73%) compared with acupuncture (2%).

**Table 1:** Frequency of overall response groups during acupuncture stimulation and tactile stimulation

		**Overall**	**Acupuncture**			**Tactile stimulation**			**Fisher's**
**Acupoint**	**N**	**Response**	***N***	***%***	***SE***	***N***	***%***	***SE***	***p***
***Threshold = 1***									
All	45	Deqi	33	73.3	4.0	17	37.4	1.8	0.010
		Mixed	12	26.7	6.6	1	2.2	2.2	0.002
		None	-	-	-	24	53.3	7.4	0.0001
LI4	16	Deqi	12	75.0	6.3	10	62.5	3.8	0.7
		Mixed	4	25.0	10.8	1	6.3	6.1	0.3
		None	-	-	-	5	31.3	11.6	0.04
ST36	16	Deqi	12	75.0	6.3	6	37.5	15.6	0.07
		Mixed	4	25.0	10.8	-	-	-	0.1
		None	-	-	-	10	62.5	12.1	0.0002
LV3	13	Deqi	9	69.2	8.5	4	30.8	14.1	0.1
		Mixed	4	30.8	12.8	-	-	-	0.1
		None	-	-	-	9	69.2	12.8	0.0005
***Threshold = 3***									
All	45	Deqi	32	71.1	4.1	11	24.4	1.9	0.0001
		Mixed	12	26.7	6.6	1	2.2	2.2	0.002
		None	1	2.2	2.2	33	73.3	6.6	0.0001
LI4	16	Deqi	12	75.0	6.3	6	37.5	4.9	0.07
		Mixed	4	25.0	10.8	1	6.3	6.1	0.3
		None	-	-	-	9	56.3	12.4	0.0008
ST36	16	Deqi	12	75.0	6.3	4	25.0	13.3	0.01
		Mixed	4	25.0	10.8	-	-	-	0.1
		None	-	-	-	12	75.0	10.8	0.0001
LV3	13	Deqi	8	61.5	9.5	1	7.7	13.3	0.01
		Mixed	4	30.8	12.8	-	-	-	0.1
		None	1	7.7	7.4	12	92.3	7.4	0.0001

Fisher's exact test. Occurrence of response groups *vs*. Non-occurrence of response group with two thresholds for inclusion. Inclusion in response group was based on the total sum of reported intensity of sensations for each subject thresholded either at a sum of 1 or a sum of 3. SE = Standard Error

As for the individual points, ST36 and LV3 showed significantly more *deqi *response in acupuncture than in tactile stimulation (p < 0.01); LI4 showed a similar trend (p < 0.07). In acupuncture, the *deqi *frequency was similar at LI4 and ST36 (75%), higher than at LV3 (56%). While none of the acupuncture procedures at LI4 and ST36 failed to show any *deqi *response, this was observed in 8% of the procedures at LV3. In tactile stimulation, the *deqi *response was more common at LI4 than at ST36 or LV3.

#### Frequency of sensations

**Figure 3:**
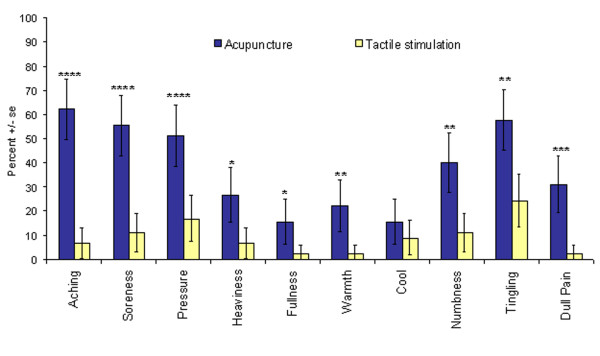
**Comparison of the frequency of different sensations between acupuncture and tactile stimulation**. When grouped across all acupoints (N = 45), virtually every sensation demonstrated a significant difference in frequency of experience between acupuncture and tactile stimulation control. In acupuncture, aching was the most frequent sensation, followed by soreness, pressure, tingling, numbness and dull pain. Tingling was the most common sensation in tactile stimulation. Error bars based on 95% Confidence Interval. Fisher's exact test: *p < 0.05, **p < 0.01, ***p < 0.001, ****p < 0.0001. For more details on data refer to Table 2.

When grouped across all acupoints, virtually every sensation demonstrated a significant difference in frequency of experience between the two conditions. The only exception of this was the cool sensation (Table 2, Figure 3). Aching led the list in the frequency of occurrence during acupuncture, followed by soreness, pressure, tingling, numbness and dull pain. Among the 10 sensations, aching stood out as the best discriminator between acupuncture and tactile control. Besides ranking first in frequency in acupuncture (62%), it rarely occurred in tactile stimulation. Its importance as a discriminator was supported by additional statistical analysis described below. Tingling and numbness, although higher in frequency than dull pain, were nevertheless less characteristic of acupuncture. They commonly occurred in tactile stimulation (tingling 24.4%, numbness 11.1%) while dull pain was rarely observed. These observations were supported by other statistical analysis described below.

**Table 2:** Comparison of frequency of sensations between acupuncture and tactile stimulation, all acupoints pooled

	**Acupuncture**			**Tactile stimulation**			**Fisher's**
**Sensations**	***N***	***%***	***SE***	***N***	***%***	***SE***	***p***
Aching	28	62.2	7.5	3	6.7	3.8	0.0001
Soreness	25	55.6	7.7	5	11.1	4.8	0.0001
Pressure	23	51.1	7.7	8	16.9	5.8	0.0001
Heaviness	12	26.7	6.8	3	6.7	3.8	0.01
Fullness	7	15.6	5.6	1	2.2	2.3	0.03
Warm	10	22.2	6.4	1	2.2	2.3	0.004
Cool	7	15.6	5.6	4	8.9	4.4	0.30
Numbness	18	40.0	7.6	5	11.1	4.8	0.002
Tingling	26	57.8	7.6	11	24.4	6.6	0.001
Dull Pain	14	31.1	7.1	1	2.2	2.3	0.0002

When grouped across all acupoints (N = 45), virtually every sensation demonstrated a significant difference in frequency of experience between acupuncture and tactile stimulation control. In acupuncture, aching was the most frequent sensation followed by soreness, pressure, tingling, numbness and dull pain. Tingling and pressure were the two most common sensations in tactile stimulation control. SE = Standard Error.

Analysis of the data on individual points demonstrated overall similarity with minor variations in the frequency of sensations. Aching, soreness, warmth and dull pain were significantly more frequent than in tactile stimulation for LI4; aching, soreness, pressure and dull pain showed significant differences for ST36; aching, soreness, pressure, warmth, numbness and tingling all showed differences for LV3 (Table 3). Dull pain was significantly more common for LI4 than for LV3 (p = 0.04). Fullness was significantly more common for LI4 than for ST36 (p < 0.05) (Table 4).

**Table 3:** Comparison of frequency of sensations between acupuncture and tactile stimulation at each acupoint

	**Acupuncture**			**Tactile stimulation**			**Fisher's**
**Sensations**	***N***	***%***	***SE***	***N***	***%***	***SE***	***p***
***LI4, N = 16***							
Aching	12	75.0	10.8	1	6.3	6.1	0.0001
Soreness	12	75.0	10.8	3	18.8	9.8	0.0019
Pressure	8	50.0	12.5	3	18.8	9.8	0.07
Heaviness	6	37.5	12.1	2	12.5	8.3	0.11
Fullness	5	31.3	11.6	1	6.3	6.1	0.09
Warm	5	31.3	11.6	-	-	-	0.02
Cool	3	18.8	9.8	2	12.5	8.3	0.5
Numbness	7	43.8	12.4	3	18.8	9.8	0.1
Tingling	11	68.8	11.6	6	37.5	12.1	0.08
Dull Pain	7	43.8	12.4	-	-	-	0.003
***ST36, N = 16***							
Aching	9	56.3	12.4	1	6.3	6.1	0.003
Soreness	7	43.8	13.8	1	6.3	6.1	0.02
Pressure	8	50.0	12.5	2	12.5	8.3	0.03
Heaviness	4	25.0	10.8	1	6.3	6.1	0.2
Fullness	-	-	-	-	-	-	-
Warm	1	6.3	6.1	1	6.3	6.1	0.8
Cool	2	12.5	8.3	1	6.3	6.1	0.5
Numbness	6	37.5	13.8	2	12.5	8.3	0.1
Tingling	8	50.0	12.5	3	18.8	9.8	0.07
Dull Pain	6	37.5	12.1	1	6.3	6.1	0.04
***LV3, N = 13***							
Aching	7	53.9	13.8	-	-	-	0.003
Soreness	6	46.2	12.5	-	-	-	0.008
Pressure	7	53.9	13.8	-	-	-	0.003
Heaviness	2	15.4	10.0	-	-	-	0.2
Fullness	2	15.4	10.0	-	-	-	0.2
Warm	4	30.8	12.8	-	-	-	0.05
Cool	2	15.4	10.0	1	7.7	7.4	0.5
Numbness	5	38.5	13.5	-	-	-	0.02
Tingling	7	53.9	13.8	2	15.4	10.1	0.05
Dull Pain	1	7.7	7.4	-	-	-	0.5

Individual points demonstrated overall similarity with minor variations in the frequency of sensations between acupuncture and tactile stimulation. Aching, soreness, warmth and dull pain were significantly more frequent in acupuncture than in tactile stimulation for LI4; aching, soreness, pressure and dull pain for ST36; aching, soreness, pressure, warmth, numbness and tingling for LV3. SE = Standard Error

**Table 4:** Comparison of frequency of sensations with acupuncture stimulation between acupoints

										**Fishers**		
										
	**LI4 (N = 16)**			**ST36 (N = 16)**			**LV3 (N = 13)**			**LI4vsST36**	**ST36vsLV3**	**LI4vsLV3**
**Sensations**	***#***	***%***	***SE***	***#***	***%***	***SE***	***#***	***%***	***SE***	***p***	***p***	***p***
Aching	12	75.0	10.8	9	56.3	12.4	7	53.9	13.8	0.5	1.0	0.3
Soreness	12	75.0	10.8	7	43.8	13.8	6	46.2	12.5	0.1	1.0	0.1
Pressure	8	50.0	12.5	8	50.0	12.5	7	53.9	13.8	1.0	1.0	1.0
Heaviness	6	37.5	12.1	4	25.0	10.8	2	15.4	10.0	0.7	0.7	0.2
Fullness	5	31.3	11.6	0	0.0	0.0	2	15.4	10.0	0.02	0.2	0.4
Warm	5	31.3	11.6	1	6.3	6.1	4	30.8	12.8	0.1	0.1	1.0
Cool	3	18.8	9.8	2	12.5	8.3	2	15.4	10.0	1.0	1.0	1.0
Numbness	7	43.8	12.4	6	37.5	13.8	5	46.2	13.8	1.0	1.0	1.0
Tingling	11	68.8	11.6	8	37.5	9.8	7	53.9	13.8	0.5	1.0	4.7
Dull Pain	7	43.8	12.4	6	37.5	12.1	1	7.7	7.4	1.0	0.09	0.04

LI4 elicited the strongest overall response of *deqi *sensations. Dull pain occurred 6 times more often in LI4 than in LV3. Fullness was significantly more frequent in LI4 than ST36. SE = Standard Error

#### Intensity of sensations

When we performed the t-tests on mean sensations only for subjects who reported a sensation above threshold in acupuncture regardless of its presence or absence in tactile stimulation control, we found a significant difference in the intensity of response for several sensations and acupoints (Table 5). In particular, aching and pressure appeared to be especially important as potential *deqi *sensations for all three acupoints (p < 0.001). Soreness, fullness, warmth, numbness, tingling, and dull pain were more intense for LI4 and ST36 (p < 0.05). Of the three acupoints, LI4 had the largest number of sensations that were significantly stronger in acupuncture than in tactile stimulation, including 7 of the 10 sensations: aching, soreness, pressure, heaviness, fullness, warmth, and dull pain. Numbness and tingling only showed a trend to be higher in acupuncture.

**Table 5:** Paired t-tests comparing sensation intensity between acupuncture and tactile stimulation, when these sensations occurred in acupuncture

	**p-value**			**N**		
				
**Sensations**	***LI4***	***ST36***	***LV3***	***LI4***	***ST36***	***LV3***
Aching	0.0000	0.0003	0.01	12	9	7
Soreness	0.01	0.5	0.05	12	7	6
Pressure	0.001	0.01	0.006	8	8	7
Heaviness	0.02	0.6	0.3	6	4	2
Fullness	0.01	--	0.03	5	0	2
Warm	0.01	--	0.01	5	1	4
Cool	0.5	--	0.06	3	2*	2
Numbness	0.07	0.00	0.02	7	6	5
Tingling	0.07	0.02	0.01	11	8	7
Dull Pain	0.003	0.05	--	7	6	1

* Not tested (variance = 0).

T-tests were performed on sensations men scores only for subjects who reported a sensation above a threshold of 1 in acupuncture regardless of its presence or absence in tactile stimulation. Aching and pressure appear to be especially important as potential *deqi *sensations for all three acupoints (p < 0.001). Soreness, fullness, warmth, numbness, tingling, and dull pain were more intense for two of the acupoints (p < 0.05). Of the three acupoints, LI4 had the largest number of sensations that were significantly stronger in acupuncture, including aching, soreness, pressure, heaviness, fullness, warmth, and dull pain.

#### Correlation of sensation intensities

**Figure 4:**
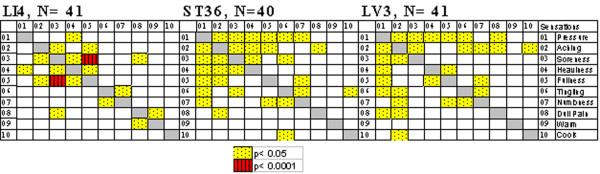
**Correlation of intensity of sensations in acupuncture (zero values are not included)**. Spearman's correlation was performed to determine if a correlation existed between the intensities of the different sensations that occurred during acupuncture for each acupoint, conditioned that the sensation was experienced at the minimal score of 1. All 3 points demonstrated positive correlations between aching and soreness, between heaviness and pressure, between dull pain and aching or soreness, and between tingling and numbness. The strongest correlation was seen between aching and fullness at LI4.

As shown in Figure 4, positive correlation was observed between the intensities of specific sensations. All three points demonstrated correlation between aching and soreness, between heaviness and pressure, between dull pain and aching or soreness, and between tingling and numbness. The strongest correlation was seen between aching and fullness at LI4. Pressure correlated with most other sensations at ST36 and LV3.

#### Specificity of individual sensations for acupuncture *vs*. tactile stimulation control

In this statistical test, we are testing for co-occurrence of the sensations in acupuncture and tactile stimulation. Thus, not finding a significant result (large p-values) indicates the occurrence of a sensation is distinct between acupuncture and tactile stimulation for each subject. This applies to almost all the sensations in the pooled data as well as from individual acupoint data. The only exception was tingling. The small p values for tingling suggest that the sensation was significantly associated with both acupuncture and tactile stimulation by the pooled data test (p = 0.03), and trending toward an association for LI4 by the individual acupoint test (p = 0.09). Since there was *a-priori *reason to suspect that tingling would be associated with tapping as well as needling (See Figure 3, Table 2), one could make the case that tingling may be of limited use in characterizing *deqi*.

#### Comparing the degree by which acupuncture exceeded tactile stimulation control in frequency and in intensity for each sensation across the acupoints

In Table 7 we can see that the degree by which acupuncture exceeded tactile stimulation in frequency and in magnitude did not show significant differences between acupoints. The only exception was dull pain, where the frequency tends to be higher for LI4 than ST36 and LV3 (Fisher's exact test, p = 0.09). This trend was further supported by the ANOVA tests on intensity. Both non-thresholded and thresholded data showed significant differences between acupoints for this sensation (middle and right columns respectively); LI4 exceeded ST36 by a wide margin (p = 0.02). Warmth was stronger in intensity at LI4 than in ST36 or LV3; no significant difference was observed in frequency.

**Table 7:** Comparison of acupuncture – tactile stimulation differences between acupoints

	**Frequency (Acup>Tactile stim. True/False)**	**Intensity**	
	***Fisher's Exact***	***ANOVA***	
	***(All data)***	***(All data)***	***(Threshold)***
**Sensations**	***p***	***p***	***p***
Aching	0.17	0.13	0.29
Soreness	0.37	0.21	0.49
Pressure	1.00	0.78	0.34
Heaviness	0.67	0.23	0.25
Fullness	0.09	0.20	0.76
Warm	0.11	0.34	0.05
Cool	1.00	0.89	0.88
Numbness	0.74	0.84	0.87
Tingling	0.61	0.47	0.55
Dull Pain	0.09	0.02	0.03

Comparison of the degree by which acupuncture exceeded tactile stimulation in frequency and in magnitude between the acupoints. No significant differences were found between the acupoints with the exception of dull pain and warmth. Analyses with thresholded data included only subjects who reported a sensation with a score of 1 or above in acupuncture.

#### Rank ordering of sensation differences

**Figure 5:**
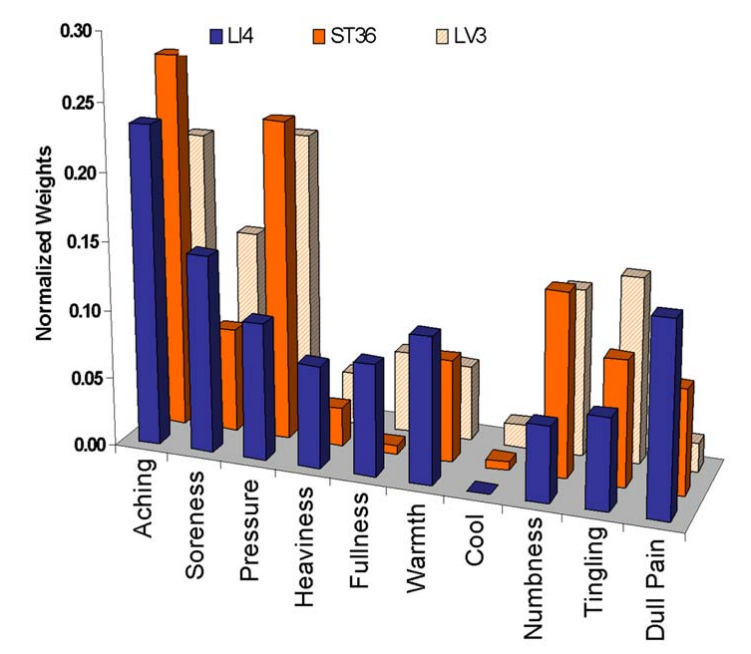
***Deq*i weights of sensations to reduce the set of sensations to a single value for each acupoint**. The sensations that are more indicative of acupuncture than tactile control have larger weights. For all three acupoints (LI4 N = 16, ST36, N = 16, LV3 N = 13), aching, soreness and pressure appeared to be more indicative of acupuncture than of tactile control stimulation. Numbness, tingling and dull pain varied in their importance for each of the acupoints. For more details on data see Table 8.

The mean of differences in intensity between acupuncture and tactile stimulation control for each acupoint, their *deqi *weights as well as the sensations ranked according to these weights, are presented in Table 8 and Figure 5. From the rankings, it can be seen that aching, soreness and pressure appeared to be important for the characterization of *deqi *for all three acupoints, coolness and heaviness were not important for any of the acupoints, while numbness, tingling and dull pain differed widely in their contribution to *deqi *for the different acupoints. Aching led the list in the rank ordering, showing the largest difference for LI4 and ST36 and the next to largest for LV3. Dull pain was most acupoint dependent, ranking second highest for LI4 and second lowest for LV3.

**Table 8:** Mean intensity differences, *deqi *weights, and associated ranking of sensations for each acupoint

	**Mean Intensity Difference (SE)**			**Deqi Weights (Ranked)**		
**Sensations**	***LI4***	***ST36***	***LV3***	***LI4***	***ST36***	***LV3***
Aching	2.75 (0.53)	1.38 (0.38)	1.66 (0.62)	0.23 (10)	0.27 (10)	0.21 (9)
Soreness	1.69 (0.59)	0.38 (0.47)	1.09 (0.50)	0.14 (8)	0.08 (5)	0.13 (7)
Pressure	1.17 (0.74)	1.17 (0.39)	1.72 (0.63)	0.10 (7)	0.23 (9)	0.21 (10)
Heaviness	0.88 (0.44)	0.14 (0.20)	0.31 (0.24)	0.07 (4)	0.03 (3)	0.04 (3)
Fullness	0.95 (0.53)	0.03 (0.03)	0.48 (0.33)	0.08 (5)	0.01 (1.5)	0.06 (5)
Warm	1.25 (0.53)	0.38 (0.52)	0.44 (0.20)	0.11 (6)	0.07 (4)	0.05 (4)
Cool	0.00 (0.26)	0.03 (0.13)	0.13 (0.17)	0.00 (1)	0.01 (1.5)	0.02 (1)
Numbness	0.64 (0.55)	0.67 (0.55)	0.98 (0.41)	0.05 (2)	0.13 (8)	0.12 (6)
Tingling	0.78 (0.40)	0.46 (0.40)	1.10 (0.37)	0.07 (3)	0.09 (7)	0.14 (8)
Dull Pain	1.64 (0.60)	0.39 (0.60)	0.17 (0.17)	0.14 (9)	0.08 (6)	0.02 (2)

Comparison of the mean of differences in intensity between acupuncture and tactile stimulation for each acupoint, their *deqi *weights as well as the sensations ranked according to these weights. Aching, soreness and pressure appeared to be important for the characterization of *deqi *for all three acupoints, coolness, heaviness and warmth were not important for any of the acupoints, while numbness, tingling and dull pain differed widely in their contribution to *deqi *for the different acupoints. SE = Standard Error

#### *Deqi *composite: Differentiation of tactile stimulation and acupuncture stimulation by weighted mean intensity

As a result of this analysis, we get a better idea if the application of weight reduced the variations between acupoints in regards to the differences between acupuncture and tactile stimulation control. As seen in Table 9, most weighted scores of intensity were greater than the simple average. The largest change as a result of applying the weights was in ST36 (F-values 35.01 *vs*. 23.11, P-values 1.76 × 10^-6 ^*vs*. 4.01 × 10^-5^). When all points were pooled, the weighted average yielded a dramatic improvement compared to the simple average (F-values 50.43 *vs*. 37.45; P-values 3.04 × 10^-10 ^*vs*. 2.67 × 10^-8^). This pattern of improvement in sensitivity was also demonstrated separately for two of the three points, LI4 and ST36. The exception was LV3 when only a small decrease in sensitivity was noted. In general, use of the weighted average appeared to provide greater power in detection of the sensations specific to the *deqi *experience.

**Table 9:** Comparison of simple and weighted intensity between acupuncture and tactile stimulation

		**Acupuncture**		**Tactile stimulation**		**Acup *vs*. Tactile stim.**	
***Acupoint***	***N***	***Intensity***	***SE***	***Intensity***	***SE***	***Fstat***	***p***
***Simple Average***							
All	45	10.67	1.23	2.37	0.58	37.45	2.67 × 10^-8^
LI4	16	15.80	2.63	4.41	1.20	16.59	3.10 × 10^-4^
ST36	16	7.16	0.45	3.77	0.94	23.11	4.01 × 10^-5^
LV3	13	8.68	2.01	0.60	0.26	15.95	5.00 × 10^-4^
*Between acupoints p < 0.005*							
							
***Weighted Average***							
All	45	1.36	0.15	0.22	0.05	50.43	3.04 × 10^-10^
LI4	16	1.91	0.24	0.32	0.24	22.53	4.76 × 10^-5^
ST36	16	1.08	0.09	0.23	0.11	35.01	1.76 × 10^-6^
LV3	13	1.02	0.26	0.08	0.04	12.78	1.53 × 10^-3^
*Between acupoints p < 0.06*							

Most weighted averages of intensity were greater than the simple averages. The largest change as a result of applying the weights was in ST36. When all points were pooled, the weighted average yielded a dramatic improvement compared to the simple average. This pattern of improvement in sensitivity was also demonstrated separately for two of the three points, LI4 and ST36. Use of the weighted average appeared to provide greater power in detection of the sensations specific to the *deqi *experience. SE = Standard Error

## Discussion

Using acupuncture at several of the most commonly used acupoints, we have provided experimental evidence to support the occurrence of a unique composition of sensations termed *deqi *and that it is associated with characteristic sensations known to be conveyed by specific types of afferent nerve systems. Furthermore, this study has provided quantitative data on several basic issues, such as the percentage of the overall *deqi *response in human subjects, the types of sensations elicited, their intensities, as well as the characteristics that distinguish them from tactile control. Aching, soreness and pressure were the most common sensations for all three acupoints, followed by tingling, numbness, dull pain, heaviness, warmth, fullness and cool sensation in decreasing order. These sensations were significantly more common in acupuncture than in tactile stimulation control, with the exception of the cool sensation, which had the lowest occurrence rate. The findings are in agreement with reports in the literature [3,5,6,9].

### *Deqi *response in humans

Clinical and experimental data indicate that not all human subjects and animals respond with *deqi *and benefit from acupuncture treatments. The ratio of responders to non-responders is estimated to be 8:2 or 7:3. The individual variations may be attributed to differences in the levels of endogenous opioids and anti-opioids [10]. The finding of an overall *deqi *response of 71–73% for all acupoints combined and 62–75% for individual acupoints is consistent with the purported percentage of acupuncture responders.

### Types of sensations associated with *deqi*

Among the many components of *deqi *sensations, aching, soreness and dull pain stood out as the most important characteristics of acupuncture not only because of their high frequency in acupuncture, but also because they were very uncommon in tactile stimulation (Tables 2, 3, 4, 5, Figure 2). They also showed correlation in intensity with one another by Spearman's analysis (Figure 4). These findings are consistent with reports in the literature. Aching was found to be the most frequent sensation in manual acupuncture at ST36 [8] and at LI4 [9]. Soreness correlated with the analgesic action of acupuncture [11]. The sensations demonstrated to be characteristic of *deqi *are in accord with the list based on a survey conducted among expert acupuncturists [12], although the data were collected with the subject lying in the scanner during fMRI and not under routine clinical conditions. The convergence of findings with a study based on clinical experience lends significant support to the experimental results in this report. Importantly our decision to treat sharp pain of any form to be uncharacteristic of *deqi *receives support from this survey. Although a "dull" sensation has been described [13], this is perhaps the first report of dull pain being an important component of the *deqi *response. We found that the dull pain generally occurred in *deqi *with higher total scores, and significantly more often at LI4, the acupoint known to have the stronger general analgesic actions. It was the only sensation that was not detected in tactile stimulation. Interestingly, the temporal relationship between dull pain and sharp pain was reversed in acupuncture *deqi *compared with noxious stimulation. The dull pain described in pain literature is often referred to as 'second pain' because it generally follows sharp pain [14], while in acupuncture the dull pain occurs independently or precedes sharp pain. Moreover, repeated fMRI studies have demonstrated that the pain neuromatrix in the limbic system is deactivated by dull pain in *deqi*, in marked contrast to its activation by noxious stimulation [7,8]. Based on the characteristics of the dull pain revealed in the present study (Tables 2, 3, 4, 5, 6, 7, 8) and previous fMRI findings, the dull pain in acupuncture is distinct from the dull pain induced by noxious stimulation. The differences could be partly explained by involvement of different nerve fiber systems induced by different procedures. Most experimental studies on pain employed thermal stimulation delivered to the skin surface, while acupuncture needle manipulation delivered mechanical stimulation to nerve fibers at deeper levels.

**Table 6:** Fisher's exact for 2 × 2 classification: Tactile stimulation (true/false) *vs*. acupuncture (true/false) by subject

	**Acupoint (p-value)**			
**Sensations**	***All acupoints***	***LI4***	***LV3***	***ST36***
Aching	0.5	1.0	1.00	-
Soreness	0.3	0.5	0.44	-
Pressure	1.0	0.2	0.47	-
Heaviness	0.3	1.0	0.25	-
Fullness	1.0	1.0	-	-
Warm	1.0	-	1.00	-
Cool	0.5	0.4	1.00	1.00
Numbness	0.6	0.6	1.00	-
Tingling	0.03	0.09	1.00	0.46
Dull Pain	0.4	-	0.38	-

Not finding a significant result (large p-values) indicates that the occurrence of a sensation is distinct between acupuncture and tactile stimulation for each subject. The occurrences of all sensations with the exception of tingling were distinct between acupuncture and tactile stimulation for each subject.

### Comparison of acupoints

Many sensations were shared by the *deqi *response generated at LI4, ST36 and LV3, but more careful examination of the data revealed differences in frequency, intensity and in the weights and rank ordering of individual sensations facilitated by the *deqi composite*. Consistent with the well-known potency of LI4 in analgesic and modulatory actions, acupuncture at LI4 produced the most prominent response in terms of the overall *deqi *experience, the number of sensations elicited and their intensities, as well as the prevalence of dull pain, an important characteristic of *deqi *as mentioned earlier. The differences between acupoints applied not only to acupuncture, but also to tactile stimulation (Table 1).

The differences in the sensory experience between acupoints in both acupuncture and tactile stimulation may be related to differences in the afferent innervations between acupoints. It is known that the dorsum of the hand is highly sensitive to touch, thermal and other sensory stimuli. In addition to the faster conducting myelinated Aβ fibers involved in the transmission of touch, vibration and numbness, hairy skin is supplied with a special C fiber system that delivers an affectionate sensation or 'limbic touch' to the brain [14,19]. The gentle rhythmic tactile stimulus performed for control could also activate the tactile afferents to deliver sensations with a tranquilizing effect on the brain, although less common than acupuncture.

### *Deqi *Composite

Compared with simple averages of intensity scores, the weighted averages reduced the variation between acupoints in regard to the differences of intensity between acupuncture and tactile stimulation control. Application of the *deqi *composite will convert the complex sensation profile of *deqi *to a single value, which can then be used for more straightforward comparisons between groups of subjects, between acupoints, and between stimulation techniques. These values will be useful for correlating the *deqi *response to clinical efficacy, and by neuroimaging, to the hemodynamic response of the brain to of acupuncture in future studies.

### Relationship of sensations with nerve fiber functions

The complex sensations in *deqi *involve a wide spectrum of nerve afferents, ranging from the fast conducting, coarsely myelinated Aβ fibers with higher thresholds to the slow conducting fine unmyelinated C fibers with lower thresholds (Table 10). However, there is contention about which types of fibers play the major role in acupuncture *deqi *and acupuncture analgesia [2-6]. The results of this study suggested that the majority of the *deqi *sensations, such as aching, soreness, dull pain, and warmth involved the slower conducting Aδ and C fibers. Pressure may not be a good discriminator because it involves several nerve fiber types. Numbness and tingling, relatively common in acupuncture, involve the Aβ fibers, but they are not as specific for acupuncture as the sensations mentioned above. As a matter of fact, tingling was the most common sensation in tactile stimulation (Table 5, Figure 2). The depth at which acupuncture exerts its action locally is not completely clear. Several conjoint psychophysical, electrophysiological and histological studies in humans indicate that *deqi *first appears when the needle reaches the muscle layers [2,5,6,10]. Deep tissue afferents, but not cutaneous afferents mediated transcutaneous electrical nerve stimulation-induced antihyperalgesia [16]. However, it has been recently proposed that the more superficial connective tissue layers might be more important, based on the entwining of connective tissue around the acupuncture needle in animal models and ultrasound imaging in humans [17,18]. The results of the present study suggest that nerve fibers at all levels are involved but the deeper muscle layers with its rich supply of slow conducting fibers may play the major role.

**Table 10:** Relations of acupuncture sensations to functions of afferent nerve fibers

**Afferent Nerve Fibers**	**Diameter**	**Velocity**	Functions *	**Acupuncture Sensations (humans)**
*Group*	(μm)	(m/s)		
β II myelinated	8 – 13	40 ~ 70	touch, vibration	**numbness**
Aγ III "	4 – 8	15 ~ 40	touch, pressure	**heaviness, pressure, fullness**
Aδ III "	1 – 4	5 ~ 15	pain, warmth, cold, pressure	**soreness, pressure, pain, **warmth, cold
C IV unmyelinated	0.2 – 1	0.2 ~ 2	pain, warmth, cold, pressure autonomic postsynaptic, olfactory	**pain**, soreness, warmth, cold, pressure

*[4,14]

** [3–6, 9]

The complex pattern of sensations in the *deqi *response suggests involvement of a wide spectrum of myelinated and unmyelinated nerve fibers, particularly the slower conducting fibers in the tendinomuscular layers. The majority of the *deqi *sensations, such as aching, soreness, dull pain, and warmth involve the slower conducting Aδ and C fibers. Pressure may not be a good discriminator because it involves several nerve fiber types. Numbness and tingling, relatively common in acupuncture, involve the Aβ fibers, but they are not as specific for acupuncture as aching, soreness and dull pain that involve the Aδ and C fibers (See Table 2).

### Tactile stimulation

Most studies in the literature employ "non-point" or placebo acupuncture for controls. In this study tactile stimulation was delivered to the acupoints, not as an 'inert' control but for comparison of the response patterns. Many of the sensations comprising *deqi *in acupuncture also occurred with tactile stimulation, but at a significantly lower frequency and with a different pattern. Aching and soreness, most common in acupuncture, were rare in tactile stimulation, Tingling and numbness showed the highest frequency in tactile stimulation instead. The differences between acupuncture and tactile stimulation could be explained by the greater concentration of slower conducting Aγ, δ fibers in deeper tissue layers than in the skin [5,6]. This may also explain why as high as 73% of tactile stimulation failed to elicit *deqi *versus only 2% in acupuncture. The low incidence of *deqi *in tactile stimulation could also explain why in earlier studies with small sample sizes, the tactile stimulation control might be underpowered to demonstrate the *deqi *response and the hemodynamic response associated with it [7].

The results also demonstrated differences between acupoints in their sensory response to tactile stimulation. Similar to acupuncture, LI4, ST36 and LV3 ranked in decreasing order with regard to the percentage of *deqi *response and the number of sensations. When thresholded at a total score of 3, *deqi *was as common as 37.5% at LI4 and only 7.7% at LV3, with ST36 at an intermediate level. The dorsum of the hand is known to be richer in afferent innervations and more sensitive to touch, thermal and other stimuli than the leg or foot. It could be supplied with the C tactile afferents that deliver a 'limbic' touch to the brain [15,19]. One might speculate that the gentle rhythmic touch employed for tactile stimulation control could activate similar nerve fiber systems to produce a pleasant and tranquilizing effect on the brain.

### Limitations and suggestions for future research

One challenge common to most acupuncture research is in the design of a valid control. Since the distribution of nerve structures is ubiquitous, the minimal, superficial, sham, non-point or placebo acupuncture often employed in acupuncture research cannot be interpreted as inert controls [19]. We opted to use tactile stimulation at the acupoint, not for inert control, but for comparison with acupuncture. It would be desirable to compare the response at a 'non-acupuncture point" with classical acupoints in future studies; this was not performed because of time limitations Based on our results, an overlap of sensations between the classical acupoint and the non-point could be anticipated, depending on their tissue types and the distribution of afferent fibers and sensory receptors. It is reported that acupuncture at a non-meridian point elicited *deqi *sensations similar to those elicited at two classical acupoints, GB 37 and UB 60 [24]. In this study the sensations interview was conducted after a ten minute fMRI scan, within which the subjects had received two sets of two minutes of stimulation, not under typical clinical settings. Applications of these results to clinical settings warrant further investigation. To avoid bias on the part of the acupuncturist, the interview was conducted by another research staff on site. An alternative method would be projecting the questions onto a screen that the subject can view and provide answers by typing on a device while positioned in the scanner. However, the subject would be deprived of opportunities to clarify understanding of the questions and provide more detailed description of the sensations. The grading of the intensities of the individual sensations as well as the setting of a threshold for the *deqi *response is somewhat subjective. There is at present no reliable method to quantify any of the *deqi *sensations; it has to depend on subjective perceptions reported by the subject. We have set two thresholds of different astringency for analysis with different purposes. Importantly, the findings are specific to the manual acupuncture technique adopted and to acupoints located in muscle layers, the tissue type to which most acupoints belong. Other techniques such as electroacupuncture and acupoints located in the scalp, perisosteum and other types of tissues with different innervations would require additional investigation.

## Conclusion

In conclusion, the quantitative as well as qualitative characterization of the sensations associated with acupuncture and their correlations with the known functions of nerve fibers provide evidence in support of the *deqi *phenomenon, a concept of fundamental importance in TCM. The sensations are significantly more common in acupuncture than in tactile stimulation control, with aching, soreness and pressure leading the list for all three acupoints. The prevalence and intensities of individual sensations show differences between acupoints, with LI4 showing the strongest overall response. The '*deqi *composite' is an approach proposed for reducing the complex sensation profile of *deqi *to a single value, which will facilitate more straightforward comparisons between groups of subjects, between acupoints, and between stimulation techniques. As future work, we will correlate this composite to the hemodynamic response of the brain in the same cohort as evidenced by fMRI

## Competing interests

The author(s) declare that they have no competing interests.

## Authors' contributions

KKSH, as Principal Investigator, was responsible for the conception, design, and overseeing the implementation of the study. She drafted and finalized the manuscript. EEN participated in the coordination, statistical analyses, and revision of the manuscript for submission. MGV performed the more advanced statistical analyses and helped to draft the manuscript. JL, as licensed acupuncturist performed the acupuncture in the entire study. He also participated in the conception and design of the study. OM participated in the data collection, data analysis, and critical review of the manuscript. VN participated in data acquisition and critical review of the manuscript. SMH participated in review of the statistical analyses and the format of the manuscript. BRR, as Director of the Center, provided crucial support for pilot studies before funding was available. As Principal Investigator of the Program Project Grant for acupuncture at MGH, he provided advice for the implementation of the study and manuscript submission. NM contributed substantially to the preparation of the manuscript, including co-ordination, drafting and critical review. DNK provided advice on the statistical analyses and data interpretation. He helped in the drafting and finalizing of the manuscript. All authors have read and approved the final version of the manuscript.

## Pre-publication history

The pre-publication history for this paper can be accessed here:


